# Role of cuproptosis in mediating the severity of experimental malaria-associated acute lung injury/acute respiratory distress syndrome

**DOI:** 10.1186/s13071-024-06520-1

**Published:** 2024-10-19

**Authors:** Xinpeng Hou, Tingting Zhou, Qi Wang, Pinru Chen, Min Zhang, Lirong Wu, Wenbin Liu, Xiaobao Jin, Zhenlong Liu, Hua Li, Bo Huang

**Affiliations:** 1 Guangdong Provincial Key Laboratory of Pharmaceutical Bioactive Substances, Guangdong Pharmaceutical University, Guangzhou, 510006 People’s Republic of China; 2 School of Pharmacy, Guangdong Pharmaceutical University, Guangzhou, 510006 People’s Republic of China; 3 Guangzhou Chest Hospital, Guangzhou, 510095 People’s Republic of China; 4 Division of Experimental Medicine, Department of Medicine, McGill University, Montreal, QC Canada; 5 Department of Critical Care Medicine, Shenzhen Bao’an District Songgang People’s Hospital, Shenzhen, 518105 China; 6 School of Basic Medical Science, Guangdong Pharmaceutical University, Guangzhou, 510006 People’s Republic of China

**Keywords:** Malaria, ALI/ARDS, Cuproptosis, Macrophage, M1/M2 polarization

## Abstract

**Background:**

Malaria-associated acute lung injury/acute respiratory distress syndrome (MA-ALI/ARDS) is a fatal complication of *Plasmodium falciparum* infection that is partially triggered by macrophage recruitment and polarization. As reported, copper exposure increases the risk of malaria infection, and copper accumulation-induced cuproptosis triggers M1 macrophage polarization. It is thus hypothesized that cuproptosis could act as a critical mediator in the pathogenesis of MA-ALI/ARDS, but its underlying mechanism remains unclear. The present study aimed to explore the role of cuproptosis in the severity of murine MA-ALI/ARDS.

**Methods:**

We utilized an experimental model of MA-ALI/ARDS using female C57BL/6 mice with *P. berghei* ANKA infection, and treated these animals with the potent copper ion carrier disulfiram (DSF) or copper ion chelator tetrathiomolybdate (TTM). The RAW 264.7 macrophages, which were stimulated with infected red blood cells (iRBCs) in vitro, were also targeted with DSF-CuCl_2_ or TTM-CuCl_2_ to further investigate the underlying mechanism.

**Results:**

Our findings showed a dramatic elevation in the amount of copper and the expression of SLC31A1 (a copper influx transporter) and FDX1 (a key positive regulator of cuproptosis) but displayed a notable reduction in the expression of ATP7A (a copper efflux transporter) in the lung tissue of experimental MA-ALI/ARDS mice. Compared to the *P. berghei* ANKA-infected control group, mice that were administered DSF exhibited a remarkable increase in parasitemia/lung parasite burden, total protein concentrations in bronchoalveolar lavage fluid (BALF), lung wet/dry weight ratio, vascular leakage, and pathological changes in lung tissue. Strikingly, the experimental MA-ALI/ARDS mice with DSF treatment also demonstrated dramatically elevated copper levels, expression of SLC31A1 and FDX1, numbers of CD86^+^, CD68^+^, SLC31A1^+^-CD68^+^, and FDX1^+^-CD68^+^ macrophages, and messenger RNA (mRNA) levels of pro-inflammatory cytokines (tumor necrosis factor [TNF-α] and inducible nitric oxide synthase [iNOS]) in lung tissue, but showed a remarkable decrease in body weight, survival time, expression of ATP7A, number of CD206^+^ macrophages, and mRNA levels of anti-inflammatory cytokines (transforming growth factor beta [TGF-β] and interleukin 10 [IL-10]). In contrast, TTM treatment reversed these changes in the infected mice. Similarly, the in vitro experiment showed a notable elevation in the mRNA levels of SLC31A1, FDX1, CD86, TNF-α, and iNOS in iRBC-stimulated RAW 264.7 cells targeted with DSF-CuCl_2_, but triggered a remarkable decline in the mRNA levels of ATP7A, CD206, TGF-β, and IL-10. In contrast, TTM-CuCl_2_ treatment also reversed these trends in the iRBC-stimulated RAW 264.7 cells.

**Conclusions:**

Our data demonstrate that the activation of cuproptosis with DSF aggravated the severity of MA-ALI/ARDS by partially inducing M1 polarization of pulmonary macrophages, while inhibition of cuproptosis with TTM contrarily ameliorated the severity of MA-ALI/ARDS by promoting macrophage M2 polarization. Our findings suggest that blockage of cuproptosis could be a potential therapeutic strategy for treatment of MA-ALI/ARDS.

**Graphical Abstract:**

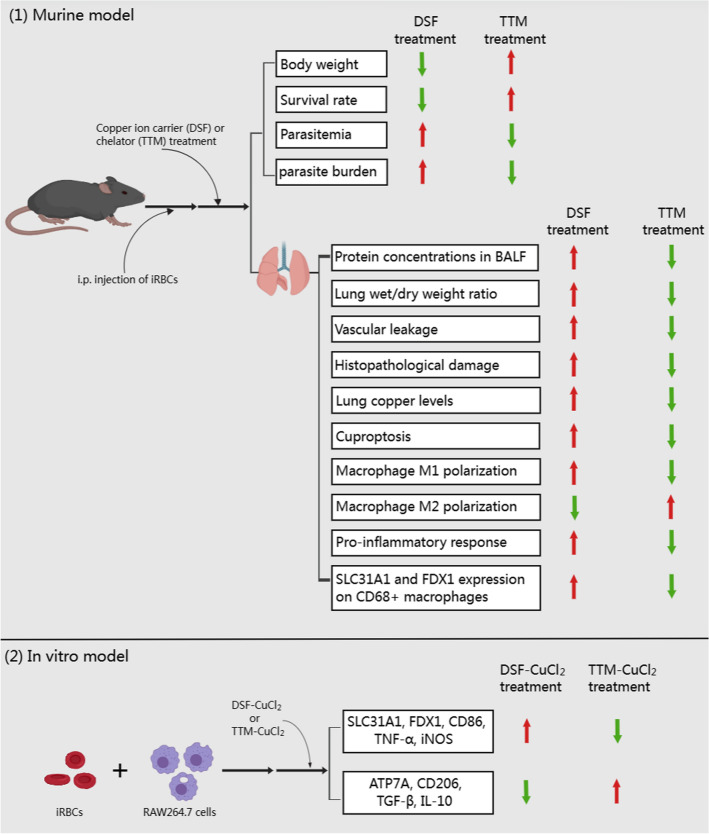

**Supplementary Information:**

The online version contains supplementary material available at 10.1186/s13071-024-06520-1.

## Background

Malaria is a mosquito-borne parasitic disease with serious implication; in 2022, approximately 249 million people were infected with the disease, leading to approximately 627,000 deaths, with most malaria cases and deaths occurring in sub-Saharan Africa [1]. *Plasmodium* spp. infection causes a complex of clinical symptoms, ranging from asymptomatic or non-specific symptoms (cyclical fevers, sweating, and chills) to severe complications (anemia, acidosis, renal failure, lung injury, cerebral malaria) and even death [2, 3]. Moreover, the syndromes of malaria-associated acute lung injury (MA-ALI) also vary between cases, from minor (cough) to symptomatic (respiratory distress, pulmonary edema), and even the most severe form (acute respiratory distress syndrome [ARDS]) [4]. Clinical studies demonstrated that ~25% of adults and ~40% of children with malaria experienced pulmonary complications and ALI/ARDS even after effective antimalarial drug treatment [5]. Of note, among patients with severe *Plasmodium falciparum* malaria who developed ALI/ARDS syndromes, the mortality rate was ~80% [6]. Typical pathological features used for characterization of MA-ALI/ARDS include sequestration of infected red blood cells (iRBCs) on lung microvasculature, infiltration/recruitment of neutrophils and macrophages, uncontrolled pro-inflammatory response, lung barrier disruption, and pulmonary edema [7, 8]. Given the limitations in accessing lung tissues from MA-ALI/ARDS patients, an experimental model of MA-ALI/ARDS using C57BL/6 mice with *P. berghei* ANKA strain, which exhibited similar clinical syndromes as MA-ALI/ARDS patients [8], was adopted to elucidate its pathological mechanisms [9].

Apart from its role as a vital organ for gaseous exchange in mammals, the lung also functions as a primary immune organ which triggers the resident innate/adaptive immune cells to induce a potent innate/adaptive immune response to pulmonary pathogen infections. Pulmonary macrophages, including tissue-resident alveolar/interstitial macrophages and recruited macrophages, are a pivotal innate immune cell with the ability for phagocytosis [10]. Macrophages are commonly divided into two functional subtypes: classically activated pro-inflammatory M1 subtype and alternatively activated anti-inflammatory M2 subtype [10]. Tremendous studies have demonstrated that infiltration/recruitment of pulmonary macrophages typically plays a protective role by triggering phagocytosis of pathogens or repairing tissues, while abnormal activity of pulmonary macrophages aggravates the severity of lung diseases by inducing extensive and uncontrolled pro-inflammatory response [11, 12]. Furthermore, it was reported that M1-subtype macrophages led to lung injury by releasing massive pro-inflammatory cytokines, while M2-subtype macrophages conversely alleviated lung pathological damage or even repaired lung tissues by inducing an anti-inflammatory response [10]. Thus, it is reasonable to speculate that the multifaceted role of pulmonary macrophages in the pathological process of lung diseases could be partially determined by an imbalance in M1/M2 polarization. It was reported that the upregulated expression of sphingosine kinase 1 and sphingosine 1-phosphate receptor 3 in alveolar macrophages was closely associated with lung injury scores in *Plasmodium berghei* ANKA-infected DBA/2 mice with ALI/ARDS syndrome [13]. Moreover, a previous study showed that patients with severe *P. falciparum* malaria with pulmonary edema displayed a dramatic elevation in the number of M1-subtype pulmonary macrophages, and further indicated that the M1-subtype pulmonary macrophages functioned as a key pathological factor in the severity of MA-ALI/ARDS [14]. Similarly, our earlier study indicated that the blockage of the mechanosensitive Piezo1 channel conversely alleviated the severity of experimental MA-ALI by partially promoting pulmonary macrophage M2 polarization [15]. Thus, these studies confirm that the severity of MA-ALI/ARDS is partially determined by the imbalance in pulmonary macrophage M1/M2 polarization. So far, however, the precise mechanism is not fully elucidated.

Copper is an indispensable trace metal for supporting normal physiological processes and a variety of cellular functions, including iron mobilization, enzyme activity, mitochondrial oxidative phosphorylation, and antioxidant defense [16]. Increasing evidence has demonstrated that excessive accumulation of intracellular copper has deleterious effects and modulates the subsequent pathological process of various lung diseases [17, 18]. Recent research has revealed an innovative form of excessive accumulation of intracellular copper-induced cell death, known as cuproptosis, characterized by the aggregation of lipoylated tricarboxylic acid (TCA) cycle proteins and loss of iron–sulfur cluster proteins [19]. Although accumulating studies have highlighted the significance of copper during various microbial infections (*Leishmania donovani*, *Cryptococcus neoformans*, and *Candida albicans*) [20–24], and bioinformatics analysis has also confirmed that cuproptosis-related molecular subtypes (*PDHA1*, *PDHB*, and *MTF1*) are potentially involved in the pathogenesis of coronavirus disease 2019 [25], there is still a lack of evidence for the function of copper or cuproptosis in pathogen infections. Similarly, previous studies showed that higher levels of serum copper were observed in malaria patients or experimental models relative to those of healthy controls [24, 26], and further demonstrated that treatment with a copper chelator inhibited the parasite’s proliferation in erythrocytes [27, 28], indicating that the accumulation of intracellular copper could be strongly associated with the parasite’s proliferation. Notably, a growing body of evidence has demonstrated that cuproptosis is a regulator in the activation, differentiation, and function of macrophages, thereby modulating the subsequent pathogenesis of lung diseases [29–31]. Furthermore, it was reported that the cuproptosis-related glutaminase gene promoted copper accumulation in alveolar macrophages and inhibited macrophage activity in patients with chronic obstructive pneumonia [29]. Similarly, the cuproptosis-related gene *PDHA1* was reported to worsen outcomes in breast cancer by promoting macrophage M2-to-M1 polarization [30]. Thus, it can be reasonably inferred that cuproptosis is involved in the severity of MA-ALI/ARDS by modulating pulmonary macrophage M1/M2 polarization, but the function and potential mechanism are not fully elucidated.

In this study, we utilized an experimental model of MA-ALI/ARDS using C57BL/6 mice with *P. berghei* ANKA infection. We administered a potent copper carrier (disulfiram [DSF]) or copper chelator (tetrathiomolybdate [TTM]) to these animals to explore the function of cuproptosis in mediating the pathogenesis of MA-ALI/ARDS. In addition, *P. berghei* ANKA-infected red blood cell (iRBCs)-stimulated RAW 264.7 macrophage cells were treated with DSF-CuCl_2_ or TTM-CuCl_2_ to further investigate and verify the underlying mechanism. Our results demonstrated that treatment with DSF in the infected mice promoted pulmonary macrophage M1 polarization and ultimately aggravated the severity of MA-ALI/ARDS, while the administration of TTM conversely inhibited the pathogenesis of MA-ALI/ARDS by inhibiting macrophage M1 polarization. Our findings can aid in deepening our understanding of the role of cuproptosis in the severity of MA-ALI/ARDS, and suggest that blockage of cuproptosis is a potential therapeutic strategy for preventing MA-ALI/ARDS.

## Methods

### Mice, parasites, and ethics statement

Six-week-old female C57BL/6 mice were obtained from the Guangdong Medical Experimental Animal Center and housed under specific-pathogen-free (SPF) conditions at the Experimental Animal Center of Guangdong Pharmaceutical University with standard temperature (25 ± 1 °C), humidity (45–65%), and a 12-h light–dark cycle. The *P. berghei* ANKA-infected red blood cells (iRBCs), which were kindly provided by Professor Jianping Song from Guangzhou University of Chinese Medicine, were stored in liquid nitrogen until subsequent use. All animal experiments were conducted in accordance with the National Experimental Animal Quality Management Law and approved by the Ethics Committee of Guangdong Pharmaceutical University (no. gdpulac2023295).

### Administration of DSF or TTM in experimental MA-ALI/ARDS mice

A total of 84 animals were divided into six groups: naïve (*n* = 12), DSF (*n* = 12), TTM (n = 12), *Pb* (*n* = 16), *Pb* + DSF (*n* = 16), and *Pb* + TTM (*n* = 16). In the naïve group, each animal received daily intraperitoneal injections of 100 μl of 0.9% NaCl solution. In the DSF and TTM groups, animals received DSF (50 mg/kg, no. 86720, Sigma-Aldrich) or TTM (30 mg/kg, no. 323446, Sigma-Aldrich) dissolved in an equivalent volume of 0.9% NaCl solution, respectively. In the *Pb*, *Pb* + DSF, and *Pb* + TTM groups, each animal was intraperitoneally infected with 1.0 × 10^6^ *P. berghei* ANKA-iRBCs, and on the following day began receiving daily intraperitoneal injections of 100 μl of 0.9% NaCl solution, DSF (50 mg/kg) diluted in an equivalent volume of NaCl solution, or TTM (30 mg/kg) diluted in NaCl solution, respectively. If the *P. berghei* ANKA-infected mice displayed neurological symptoms, including paralysis, convulsions, or coma at 6–11 days post-infection (dpi), and then died within 24 h, these animals were defined as the experimental cerebral malaria (ECM) mice. The body weight and survival time in different groups were monitored daily. Peripheral blood parasitemia was determined using Giemsa-stained thin blood smears under a light microscope starting from 3 dpi.

### Analysis of protein concentration in bronchoalveolar lavage fluid

To evaluate the changes in lung vascular injury in the experimental MA-ALI/ARDS mice after DSF or TTM treatment, the protein concentrations in bronchoalveolar lavage fluid (BALF) were determined for the animals (*n* = 6/group) at 8 and 15 dpi, as described previously [15]. Briefly, after the animal was euthanized with an intraperitoneal injection of isoflurane (50 mg/kg), 1.0 ml of ice-cold phosphate-buffered saline (PBS) was instilled and aspirated into the right lung. Subsequently, the collected BALF was centrifuged at 1000×*g* for 5 min at 4 °C, and the supernatant was stored at −80 °C for further analysis. The total protein concentration of BALF was determined using a bicinchoninic acid (BCA) protein quantification kit (Beyotime, China).

### Lung wet/dry weight ratio

After animals (*n* = 6/group) were euthanized at 8 and 15 dpi, the lung tissue was immediately extracted and weighed (wet weight). The same lung tissue was incubated at 80 °C overnight and the dry weight was recorded. The degree of pulmonary edema was indicated as the ratio of lung wet weight/dry weight.

### Assessment of pulmonary vascular leakage

To assess the changes in pulmonary vascular leakage in the experimental MA-ALI/ARDS mice after DSF or TTM treatment, animals (*n* = 6/group) were intravenously injected with 200 μl of 2% Evans blue (EB) dye (no. E2129, Sigma-Aldrich) at each examination point (8 and 15 dpi). After animals were euthanized, the lung tissue was immediately weighed (wet weight) and placed in formamide at 37 °C overnight to obtain extravasation of EB dye from the tissue. The concentration of EB dye in lung tissue was determined with a spectrophotometer at 620 nm and expressed as nanograms of EB dye per milligram of lung tissue.

### Histopathological analysis of lung tissue

To assess the lung histopathological changes in the experimental MA-ALI/ARDS mice after DSF or TTM treatment, hematoxylin and eosin (H&E) staining was performed. After animals (*n* = 6/group) were euthanized at 8 and 15 dpi, the harvested lung tissue was immediately fixed in paraformaldehyde (4%) for 2 days, embedded in paraffin, and cut into 5 μm-thick slices. The slices were stained with H&E dye (no. G1005-100ML, Servicebio), observed under a Leica DM 2500B light microscope at a magnification of ×200, and analyzed for pathological changes by two blinded pathologists. The semiquantitative histopathological score was used to evaluate the degree of lung injury from more than 20 non-overlapping fields per slice for each animal as described previously [15]. Briefly, seven pathological parameters, including intra-alveolar inflammation, interstitial inflammation, alveolar septal thickening, thrombosis, alveolar edema, hemozoin deposition, and edema, were each scored on a scale of 0 to 3, as follows: 0 = absent, 1 = mild, 2 = moderate, and 3 = severe. The total pathological scores were the sum of the scores for each parameter, with a maximum score of 21.

### Rubeanic acid copper staining and ICP-MS assay for analyzing copper accumulation in lung tissue

To assess the effect of DSF or TTM on copper accumulation in lung tissue in the experimental MA-ALI/ARDS mice, rubeanic acid copper (RAC) staining was performed. After animals (*n* = 6/group) were euthanized at 8 and 15 dpi, three non-contiguous lung sections per animal were deparaffinizated with xylene, hydrated with 100%, 95%, and 70% ethanol and double-distilled water for 5 min, and then stained with rubeanic acid solution. After thorough rinsing with PBS, the sections were stained with eosin, dehydrated with xylene, and subsequently mounted with neutral resins. The positive copper granules showed a deep black-green color, captured using a light microscope at a magnification of ×200, and analyzed by two blinded researchers. The degree of positive copper granules in lung tissue was represented as the average integrated optical density (IOD) per stained area (μm^2^) (IOD/area) using Image-Pro Plus 6.0 software under a Leica DMIRE2 microscope. The concentration of copper in lung tissue was also determined by inductively coupled plasma mass spectrometry (ICP-MS; Agilent 7500cx; Agilent Technologies, CA, USA). Briefly, the harvested lung tissue was immediately digested with MOS-HNO_3_, digested with a 3:1 mixture of concentrated HNO_3_ and MOS-HClO_4_, and then dissolved in 4% of HNO_3_. The concentration of copper in lung tissue was measured in micrograms of copper per gram lung wet weight.

### Immunohistochemical staining for SLC31A1, ATP7A, FDX1, CD68, CD86, and CD206 in lung tissues

To assess the effect of DSF or TTM on pulmonary macrophage M1/M2 polarization and cuproptosis in the experimental MA-ALI/ARDS mice, immunohistochemical staining was performed by targeting SLC31A1 (a copper input transporter protein), ATP7A (a copper output transporter protein), FDX1 (a key positive regulator of cuproptosis), CD68 (a total macrophage marker), CD86 (an M1-subtype macrophage marker), and CD206 (an M2-subtype macrophage marker) in the different groups. Briefly, after routine deparaffinization and hydration, three non-contiguous lung sections per animal (*n* = 6/group) were subjected to antigen retrieval in citrate buffer at 95 °C for 8 min, and incubated with 3% H_2_O_2_ for 25 min or 10% normal goat serum for 30 min to remove endogenous peroxidase or block non-specific binding, respectively. The slices were incubated with primary antibodies [monoclonal mouse anti-SLC31A1 antibody (1:200; no. NBP2-36573; Novus Biologicals), monoclonal mouse anti-ATP7A antibody (1:100; no. MA5-27720; Thermo Fisher Scientific), polyclonal rabbit anti-FDX1 antibody (1:200; no. 12592-1-AP; Proteintech), monoclonal rabbit anti-CD68 antibody (1:200; no. 97778S; CST), monoclonal rabbit anti-CD86 antibody (1:200; no. 19589S; CST), or polyclonal rabbit anti-CD206 antibody (1:400; no. GB113497-100; Servicebio)] overnight at 4 °C. Subsequently, the slices were subjected to the secondary antibodies [goat anti-rabbit immunoglobulin G (IgG) (H + L) horseradish peroxidase (HRP) (1:200; no. S0001; Affinity) or goat anti-mouse IgG (H + L) HRP (1:200; no. S0002; Affinity)] at 37 °C for 1 h, and stained with DAB and hematoxylin. The positively stained cells with a deep brown color were photographed under a Leica DM2500B microscope at a magnification of ×400, and then analyzed by two blinded researchers. Positive expression of SLC31A1, ATP7A, or FDX1 was represented as IOD/area from more than 20 fields per slice for each animal, while that for CD68, CD86, or CD206 was taken as positively stained cells per field.

### Double immunofluorescence staining of SLC31A1^+^-CD68^+^ and FDX1^+^-CD68^+^ pulmonary macrophages

To estimate the effect of DSF or TTM on the expression of cuproptosis-related proteins (SLC31A1 and FDX1) in the pulmonary CD68^+^ macrophages in the experimental MA-ALI/ARDS mice, double immunofluorescence staining of SLC31A1^+^-CD68^+^ and FDX1^+^-CD68^+^ pulmonary macrophages was performed. After routine deparaffinization, hydration, antigen retrieval, remove of endogenous peroxidase, and blockage of non-specific binding, the slices were incubated with monoclonal mouse anti-CD68 antibody (1:200; no. GB14043-50; Servicebio), polyclonal rabbit anti-SLC31A1 antibody (1:500; no. NB100-402; Novus Biologicals), or polyclonal rabbit anti-FDX1 antibody (1:200; no. 12592-1-AP; Proteintech) overnight at 4 °C, followed by incubation with secondary antibodies [Alexa Fluor^®^ 488 goat anti-mouse IgG (1:1000; no. 4480S; CST) and CoraLite^®^ 594 goat anti-rabbit IgG (H + L) (1:200; no. SA00013-4; Proteintech)] at 37 °C for 1 h in the dark. After through washing with PBS, the slice was stained with DAPI for 10 min and recorded using the EVOS M5000 automated cell imaging system (Thermo Fisher Scientific, USA). The CD68^+^-positive cells appeared green, while SLC31A1^+^ or FDX1^+^-positive cells were red. Co-expression of SLC31A1^+^-CD68^+^ or FDX1^+^-CD68^+^ cells appeared yellow. The number of SLC31A1^+^-CD68^+^ or FDX1^+^-CD68^+^ cells per field was determined from more than 20 fields/slice for each mouse (*n* = 6/group).

### In vitro co-culture of iRBC-stimulated RAW 264.7 macrophages with DSF-CuCl_2_ or TTM-CuCl_2_ treatment

Murine RAW 264.7 macrophages were kindly provided by the stem cell bank of the Chinese Academy of Sciences (Shanghai, China) and cultured in Dulbecco’s modified Eagle's medium (DMEM) containing 6% fetal bovine serum (FBS; no. A2720801, Gibco™) and 1% penicillin/streptomycin at 37 °C 5% and CO_2_. RAW 264.7 cells (2.0 × 10^5^/well) were seeded in six-well plates for 6 h and then stimulated or not with iRBCs (5.0 × 10^6^/well), followed by the addition of 100 μl of a sterile PBS buffer containing CuCl_2_ (10 μM), DSF (20 nΜ) + CuCl_2_ (10 μM), or TTM (20 nΜ) + CuCl_2_ (10 μM) for 24 and 48 h. After thorough washing with cold PBS, the RAW267.4 cells pellets were harvested and stored in TRIzol reagent (no. 9109, TaKaRa Bio) at −80 °C until use.

### Quantitative polymerase chain reaction (qPCR) analysis

Total RNA was extracted from lung tissue or RAW267.4 cells in the various groups using TRIzol reagent according to the manufacturer's protocol. The total RNA (1000 ng) was reverse-transcribed into complementary DNA (cDNA) using the PrimeScript™ 1st Strand cDNA Synthesis Kit (no. 6210B, TaKaRa Bio). Subsequently, the relative expression of targeted genes was calculated using SYBR Green qPCR Master Mix (no. 6110B, TaKaRa Bio, Japan) and analyzed using the 2^−ΔΔCT^ method. The primers used for the qPCR were designed by Sangon Biotech (Shanghai, China) and are listed in Additional file 1: Table S1. The qPCR reactions consisted of 95 °C for 1 min, and 40 amplification cycles of 95 °C for 5 s and 60 °C for 20 s.

### Statistical analysis

Data were statistically analyzed using GraphPad Prism 8.0 software and are presented as mean ± standard deviation (SD). Differences between two groups or among three or more groups were determined by independent-samples *t*-tests or one-way analysis of variance (ANOVA), respectively. The significance of differences in body weight, parasitemia, or survival time among different groups was analyzed using the log-rank test or time-series analysis, respectively. A *P*-value less than 0.05 was considered statistically significant.

## Results

### Changes in morbidity and mortality in *P. berghei* ANKA-infected mice after DSF or TTM treatment

**Fig. 1:**
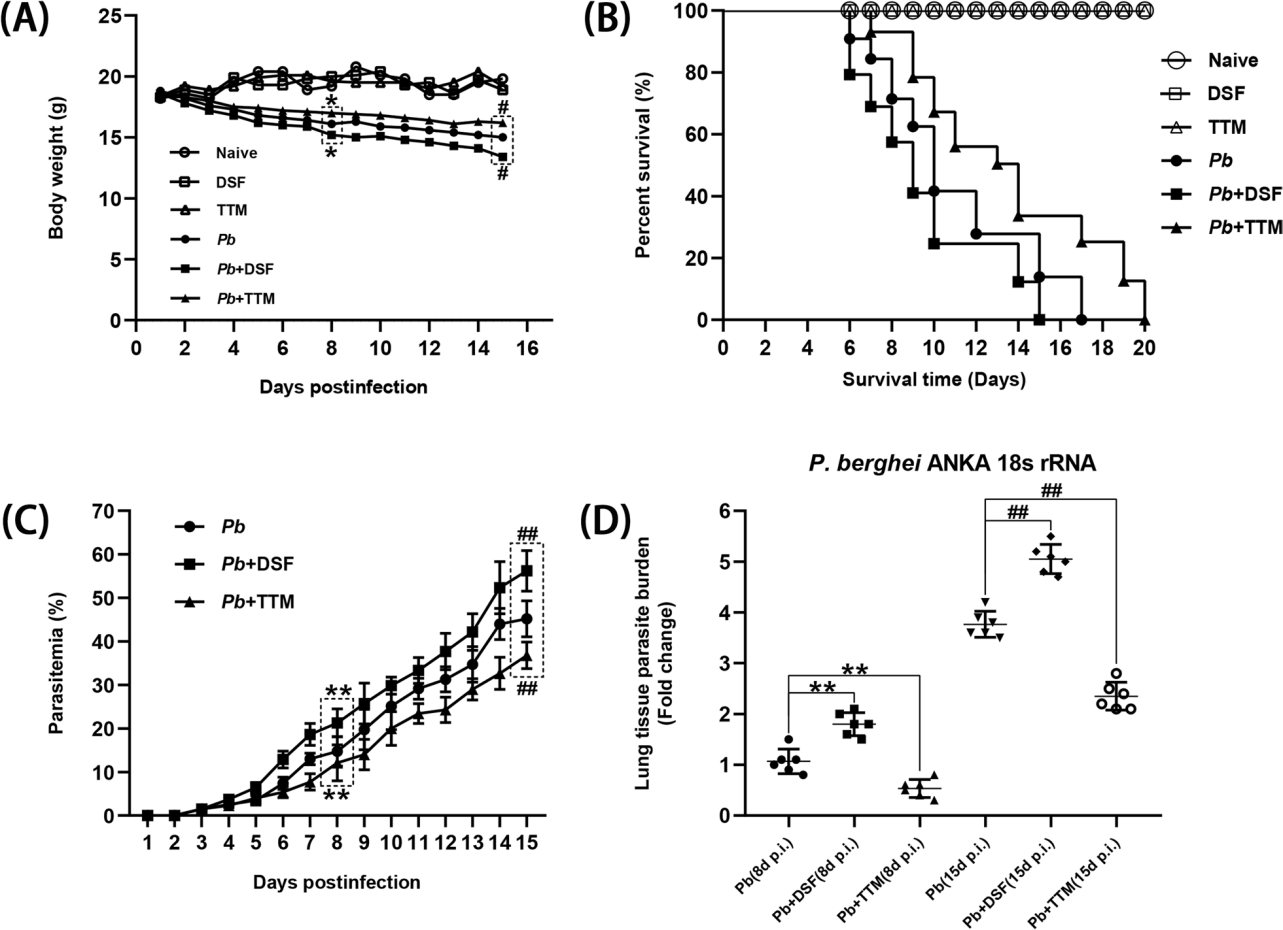
The effect of DSF or TTM on morbidity and mortality in response to *P. berghei* ANKA infection. **A**, **B** Body weight and survival time were monitored daily in the different groups and analyzed by time-series analysis and log-rank test, respectively. **C** Peripheral blood parasitemia levels were determined in the different groups by Giemsa-stained thin blood smears. Differences in parasitemia were compared using a time-series analysis. **D** Estimation of lung parasite burden at 8 and 15 dpi was determined by calculating the mRNA levels of *P. berghei* ANKA 18S rRNA with qPCR assay. *P < 0.05 and **P < 0.01 vs. the *P. berghei* ANKA-infected control mice at 8 dpi; ^#^*P* < 0.05 and ^##^*P* < 0.01 vs. the *P. berghei* ANKA-infected control mice at 15 dpi

To assess the effect of DSF or TTM on morbidity and mortality in response to *P. berghei* ANKA infection, the body weight, survival time, and peripheral blood parasitemia/lung parasite burden were monitored daily (Fig. 1). Over the 15 days of observation, no significant changes in body weight from the initial weight were observed in the uninfected mice in the naïve, DSF, or TTM group (*P* > 0.05), while the *P. berghei* ANKA-infected control mice (*Pb* group) showed a remarkable decline in body weight at 4–15 dpi (*P* < 0.05; Fig. 1A). In addition, the *P. berghei* ANKA-infected control mice in the *Pb*A group showed ~12% and ~18% loss relative to the initial body weight at 8 and 15 dpi, respectively. Remarkably, the infected mice with DSF treatment showed a higher body weight loss than those of the *Pb* group (~16% vs. ~12% at 8 dpi, *P* < 0.05; ~24% vs. ~18% at 15 dpi, *P* < 0.05), whereas those administered TTM displayed a lower body weight loss than those of the *Pb* group (~8% vs. ~12% at 8 dpi, *P* < 0.05; ~15% vs. ~18% at 15 dpi, *P* < 0.05, respectively). As shown in Fig. 1B, the *P. berghei* ANKA-infected control mice were moribund within 6–17 dpi, during which ~57% of mice developed into ECM mice. However, the infected mice with DSF treatment died at 6–15 dpi and showed a higher incidence of ECM (~70%, *P* < 0.05), while the infected mice with TTM treatment died at 7–20 dpi and exhibited a lower incidence of ECM (~45%, *P* < 0.05), than those of the *Pb* group. As shown in Fig. 1C, higher peripheral blood parasitemia levels were found in the infected animals in the *Pb* + DSF group than those in the *Pb* group at 5–15 dpi, but a lower peripheral blood parasitemia was observed in the *Pb* + TTM group. When compared with the *Pb* group at 8 and 15 dpi, the lung parasite burden was dramatically elevated in the infected mice in the *Pb* + DSF group (*P* < 0.01), but levels were notably decreased in the *Pb* + DSF group (*P* < 0.01), as determined by calculating the mRNA levels of parasite 18S ribosomal RNA (rRNA) (Fig. 1D). Thus, our findings indicate that DSF treatment exacerbated body weight loss, ECM incidence, and parasitemia/lung parasite burden, and shortened survival time in the *P. berghei* ANKA-infected mice, while TTM ameliorated morbidity and mortality in response to *P. berghei* ANKA infection.

### Changes in lung injury in experimental MA-ALI/ARDS mice after DSF or TTM treatment

**Fig. 2:**
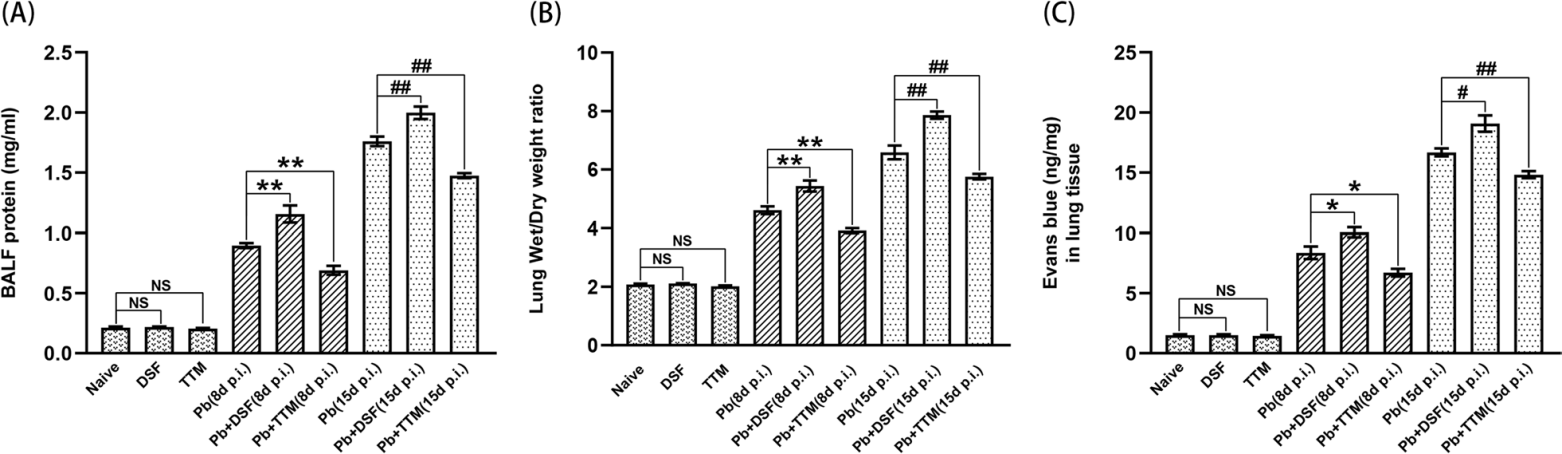
The effect of DSF or TTM on changes in total protein concentration in BALF, lung wet/dry weight ratio, and lung vascular leakage in the experimental MA-ALI/ARDS mice. **A** Total protein concentration in BALF was determined in different groups using a BCA protein quantification kit. **B** The degree of pulmonary edema was assessed by calculating the ratio of lung wet/dry weight. **C** The degree of lung vascular leakage was determined by calculating the concentration of Evans blue dye in lung tissue. Differences between two groups or among multiple groups were compared using the independent-samples *t*-test or one-way ANOVA, respectively. Experiments were conducted with six mice per group, and data are presented as mean ± SD. *P < 0.05 and **P < 0.01 vs. the *P. berghei* ANKA-infected control mice at 8 dpi; ^#^*P* < 0.05 and ^##^*P* < 0.01 vs. the *P. berghei* ANKA-infected control mice at 15 dpi. NS = non-significant, *P* > 0.05, relative to naïve mice

To evaluate the changes in lung injury in the experimental MA-ALI/ARDS mice after DSF or TTM treatment, MA-ALI/ARDS-related determinants, including total protein concentration in BALF, lung wet/dry weight ratio, and lung vascular leakage, were analyzed. As shown in Fig. 2, treatment with DSF or TTM did not induce significant changes in these three determinants among the naïve, DSF, and TTM groups. However, the *P. berghei* ANKA-infected control mice exhibited higher total protein concentration in BALF (*P* < 0.01), lung wet/dry weight ratio (*P* < 0.01), and lung EB dye amount (*P* < 0.01) at 8 and 15 dpi relative to levels in naïve mice. Strikingly, when compared with the *Pb* group, treatment with DSF led to a dramatic increase in total protein concentration in BALF (*P* < 0.01), lung wet/dry weight ratio (*P* < 0.01), and lung EB dye amount (*P* < 0.05) in the lung tissue at 8 and 15 dpi, while administration of TTM led to a remarkable decrease in these three determinants in experimental MA-ALI/ARDS mice.

**Fig. 3:**
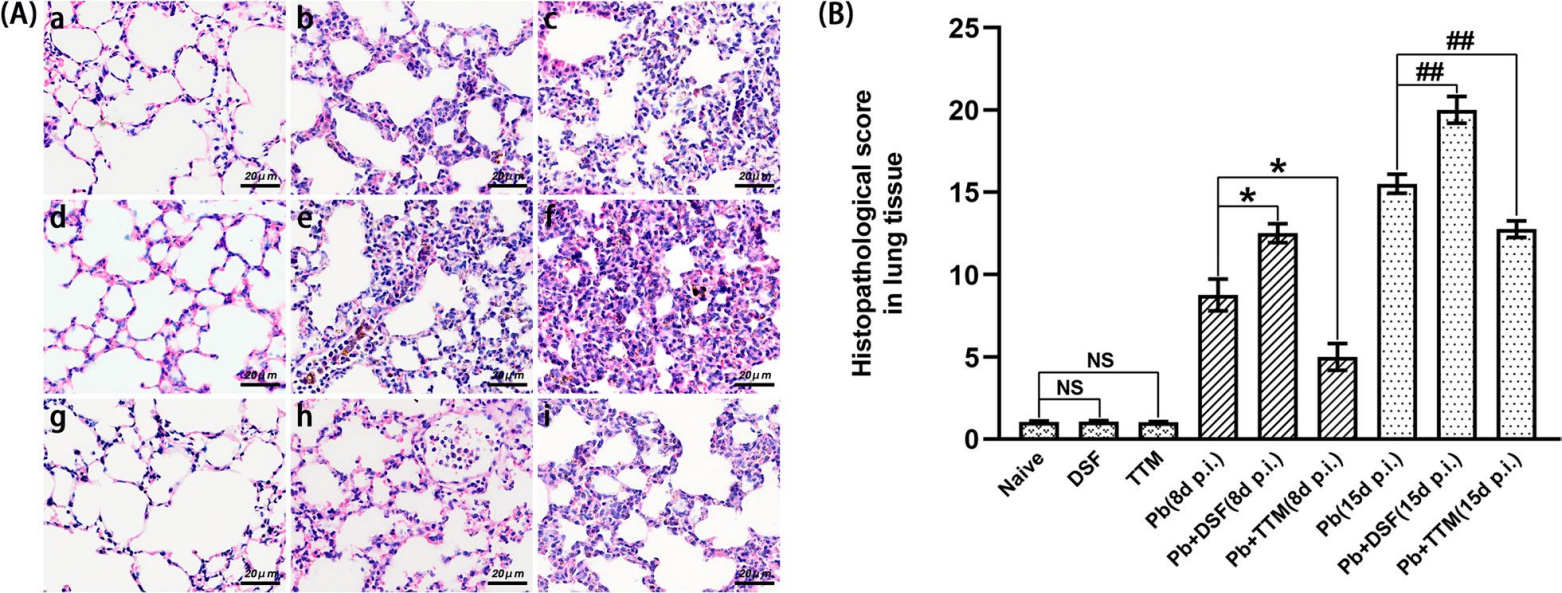
The effect of DSF or TTM on lung histopathological changes in the experimental MA-ALI/ARDS mice after DSF or TTM treatment. **A** Representative images of lung histopathological changes by H&E staining under a light microscope at a magnification of ×200: **a** naïve mice; **b** *P. berghei* ANKA-infected control mice at 8 dpi; **c** *P. berghei* ANKA-infected control mice at 15 dpi; **d** DSF-treated uninfected mice; **e** DSF-treated *P. berghei* ANKA-infected mice at 8 dpi; **f** DSF-treated *P. berghei* ANKA-infected mice at 15 dpi; **g** TTM-treated uninfected mice; **h** TTM-treated *P. berghei* ANKA-infected mice at 8 dpi; **i** TTM-treated *P. berghei* ANKA-infected mice at 15 dpi. **B** Analysis of semiquantitative lung histopathological scores. Differences in semiquantitative histopathological scores between two groups or among multiple groups were compared using the independent-samples *t*-test or one-way ANOVA, respectively. Experiments were conducted with six mice per group, and data are presented as mean ± SD. *P < 0.05 and **P < 0.01 vs. the *P. berghei* ANKA-infected control mice at 8 dpi; ^#^ *P* < 0.05 and ^##^*P* < 0.01 vs. the *P. berghei* ANKA-infected control mice at 15 dpi. NS = non-significant, *P* > 0.05, relative to naïve mice

HH&E staining showed rare or no obvious morphological or pathological changes in the lung tissue of the uninfected mice in the naïve, DSF, and TTM groups (Fig. 3A). However, the *P. berghei* ANKA-infected control mice clearly exhibited multiple pathological characteristics in the lung tissues at 8 and 15 dpi, including iRBCs, malarial pigment, infiltration of inflammatory cells, thickening of alveolar walls, pulmonary edema, and pulmonary thrombosis. When compared with the *Pb* group, the mice administered DSF demonstrated dramatically exacerbated lung histopathological features and higher semiquantitative scores at 8 dpi (*P* < 0.05) and 15 dpi (*P* < 0.01), while treatment with TTM conversely alleviated lung histopathological changes and reduced semiquantitative scores (*P* < 0.05 or *P* < 0.01), respectively. Taken together, our findings indicate that treatment with DSF or TTM exacerbated or improved lung injury in the process of MA-ALI/ARDS, respectively.

### Changes in the level of copper and the expression of cuproptosis-related proteins in the lung tissue of experimental MA-ALI/ARDS mice after DSF or TTM treatment

**Fig. 4:**
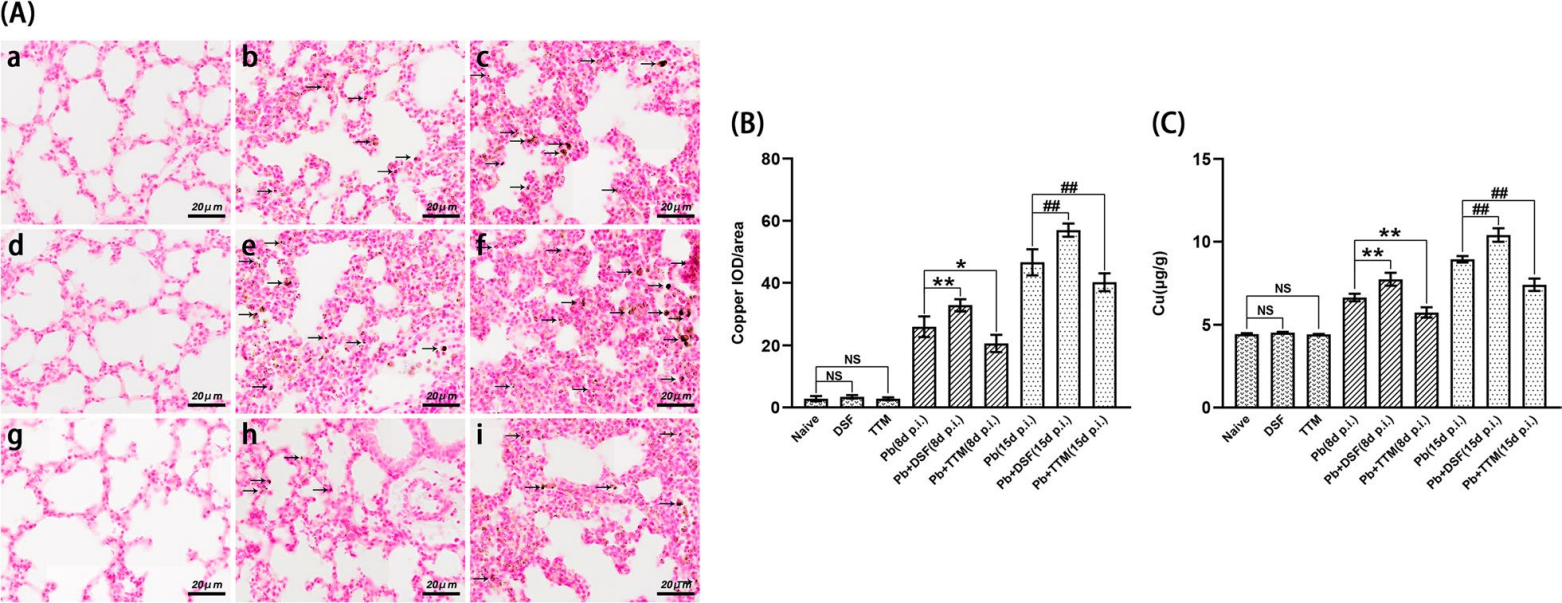
The effect of DSF or TTM on pulmonary copper accumulation in the experimental MA-ALI/ARDS mice. **A** Representative images of pulmonary copper accumulation in mice using rubeanic acid copper staining under a light microscope at a magnification of ×200. The positive copper granules show a dark brown color (arrows). **a** Naïve mice; **b** *P. berghei* ANKA-infected control mice at 8 dpi; **c** *P. berghei* ANKA-infected control mice at 15 dpi; **d** DSF-treated uninfected mice; **e** DSF-treated *P. berghei* ANKA-infected mice at 8 dpi; **f** DSF-treated *P. berghei* ANKA-infected mice at 15 dpi; **g** TTM-treated uninfected mice; **h** TTM-treated *P. berghei* ANKA-infected mice at 8 dpi; **i** TTM-treated *P. berghei* ANKA-infected mice at 15 dpi. **B** The degree of positive copper granules in lung tissue was analyzed by IOD/area using Image-Pro Plus 6.0 software under a Leica DMIRE2 microscope at a magnification of ×200. **C** The concentration of copper in lung tissue was determined by ICP-MS. Differences in the degree of positive copper granules and concentration of copper between two groups or among multiple groups were compared using the independent-samples *t*-test or one-way ANOVA, respectively. Experiments were conducted with six mice per group, and data are presented as mean ± SD. *P < 0.05 and **P < 0.01 vs. the *P. berghei* ANKA-infected control mice at 8 dpi; ^#^*P* < 0.05 and ^##^*P* < 0.01 vs. the *P. berghei* ANKA-infected control mice at 15 dpi. NS = non-significant, *P* > 0.05, relative to naïve mice

As shown in Fig. 4, RAC staining showed only slight or no pulmonary copper salt accumulation in the uninfected mice in the naïve, DSF, and TTM groups, while the degree of pulmonary copper salt accumulation was progressively elevated in the *P. berghei* ANKA-infected control mice at 8 and 15 dpi (*P* < 0.01). Notably, treatment with DSF in the infected mice dramatically increased the pulmonary copper salt accumulation at 8 and 15 dpi (*P* < 0.01) whereas the administration of TTM significantly reduced accumulation (*P* < 0.05 and *P* < 0.01) in the infected mice, respectively. Similar trends in pulmonary copper concentrations were observed with DSF or TTM treatment in the experimental MA-ALI/ARDS mice using ICP-MS (Fig. 4C).

**Fig. 5:**
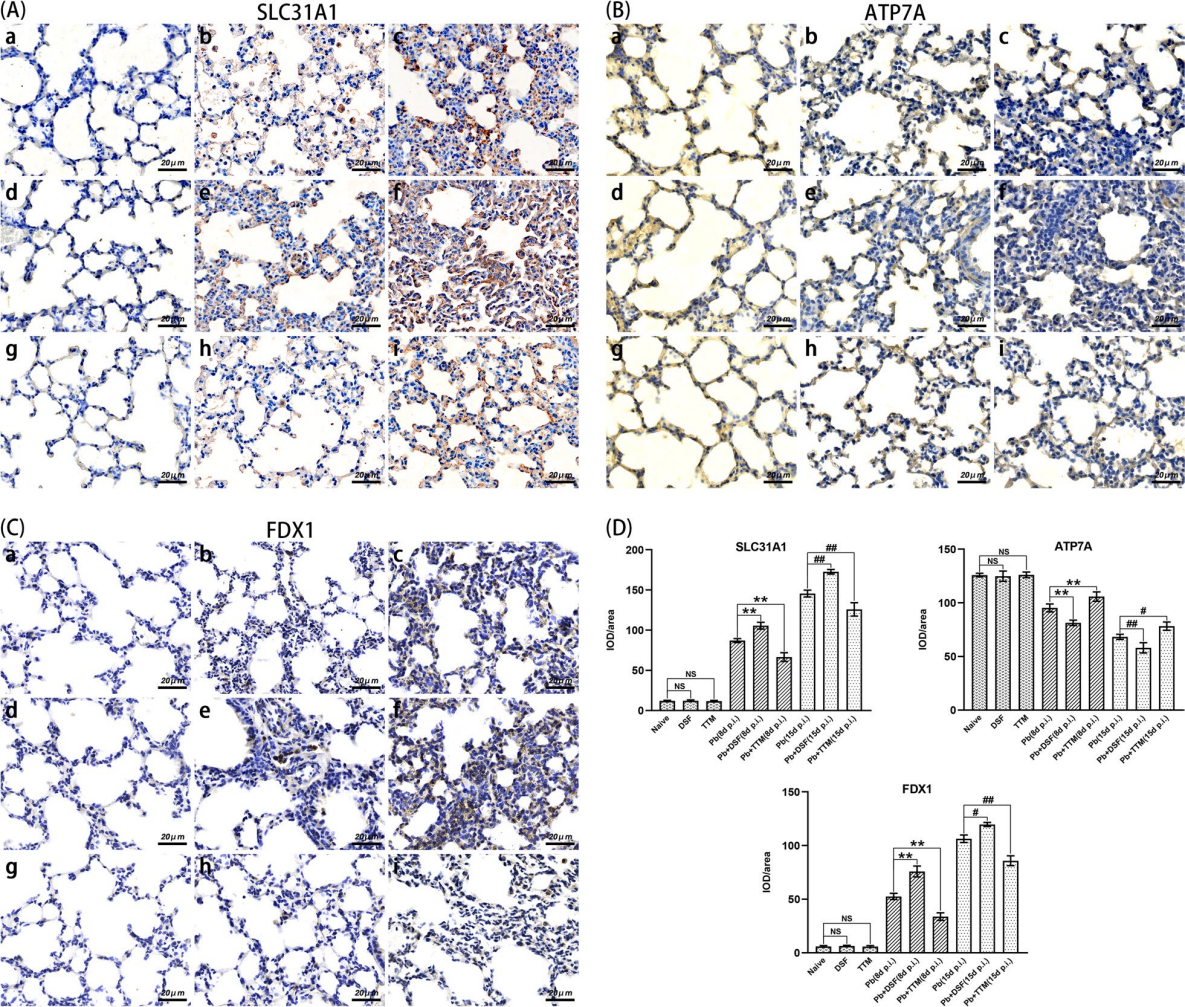
The effect of DSF or TTM on pulmonary cuproptosis-related marker expression in the experimental MA-ALI/ARDS mice.** A–C** Representative images of pulmonary cuproptosis-related marker (SLC31A1, ATP7A, and FDX1) expression in different groups using immunohistochemical staining: **a** naïve mice; **b** *P. berghei* ANKA-infected control mice at 8 dpi; **c** *P. berghei* ANKA-infected control mice at 15 dpi; **d** DSF-treated uninfected mice; **e** DSF-treated *P. berghei* ANKA-infected mice at 8 dpi; **f** DSF-treated *P. berghei* ANKA-infected mice at 15 dpi; **g** TTM-treated uninfected mice; **h** TTM-treated *P. berghei* ANKA-infected mice at 8 dpi; **i** TTM-treated *P. berghei* ANKA-infected mice at 15 dpi. **D** The IOD/area of positively stained cuproptosis-related marker expression was calculated from more than 20 lung fields per animal. The differences in IOD/area of positive expression between two groups or among multiple groups were compared using the independent-samples *t*-test or one-way ANOVA, respectively. Experiments were conducted with six mice per group, and data are presented as mean ± SD. *P < 0.05 and **P < 0.01 vs. the *P. berghei* ANKA-infected control mice at 8 dpi; ^#^*P* < 0.05 and ^##^*P* < 0.01 vs. the *P. berghei* ANKA-infected control mice at 15 dpi. NS = non-significant, *P* > 0.05, relative to naïve mice

As depicted in Fig. 5, immunohistochemical staining showed that *P. berghei* ANKA infection induced a significant increase in the expression of SLC31A1 and FDX1 but a remarkable decline in the expression of ATP7A in the lung tissue of animals in the *Pb* group at 8 and 15 dpi relative to the naïve group (*P* < 0.01). However, higher expression of SLC31A1 (*P* < 0.01) and FDX1 (*P* < 0.05 and *P* < 0.01) but lower expression of ATP7A (*P* < 0.01) were observed in lung tissue in the *Pb* + DSF group relative to levels in the *Pb* group at 8 and 15 dpi. Conversely, treatment of infected mice with TTM (*Pb* + TTM group) induced lower expression of SLC31A1 (*P* < 0.01) and FDX1 (*P* < 0.01) but higher expression of ATP7A (*P* < 0.05 and *P* < 0.01) relative to levels in the *Pb* group. Thus, our findings indicate that pulmonary cuproptosis can occur in the process of MA-ALI/ARDS, and further demonstrate that treatment with DSF or TTM could exacerbate or mitigate pulmonary cuproptosis in MA-ALI/ARDS mice, respectively.

### Changes in pulmonary macrophage M1/M2 polarization in experimental MA-ALI/ARDS mice after DSF or TTM treatment

**Fig. 6:**
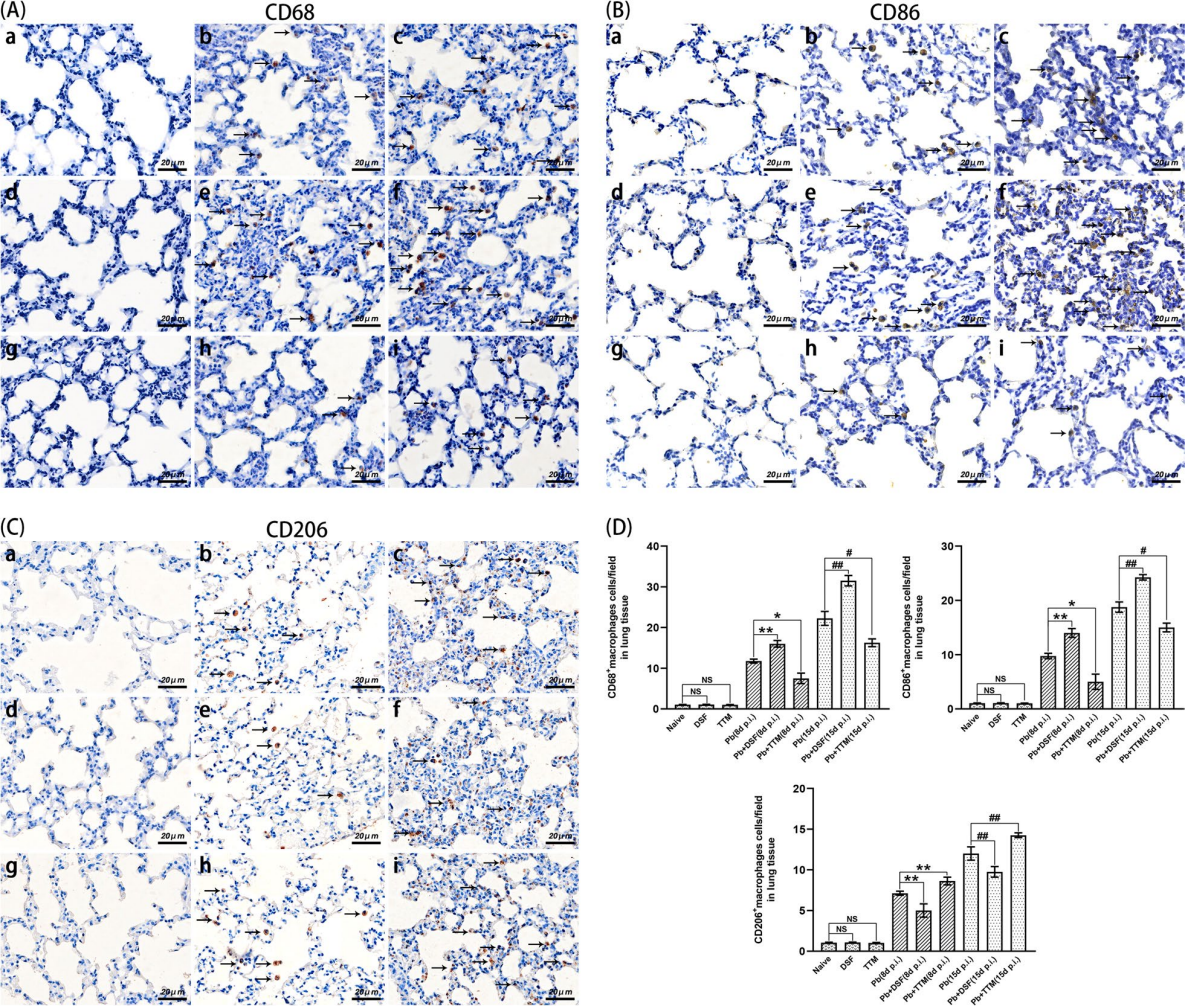
The effect of DSF or TTM on pulmonary macrophage M1/M2 polarization in experimental MA-ALI/ARDS mice.** A**–**C** Representative images of positively stained pulmonary CD68^+^, CD86^+^, and CD206^+^ macrophages in different groups using immunohistochemical staining. The positively stained CD68^+^, CD86^+^, and CD206^+^ macrophages showed a dark brown color (arrows). **a** Naïve mice; **b** *P. berghei* ANKA-infected control mice at 8 dpi; **c** *P. berghei* ANKA-infected control mice at 15 dpi; **d** DSF-treated uninfected mice; **e** DSF-treated *P. berghei* ANKA-infected mice at 8 dpi; **f** DSF-treated *P. berghei* ANKA-infected mice at 15 dpi; **g** TTM-treated uninfected mice; **h** TTM-treated *P. berghei* ANKA-infected mice at 8 dpi; **i** TTM-treated *P. berghei* ANKA-infected mice at 15 dpi. **D** The numbers of positively stained CD68^+^, CD86^+^, and CD206^+^ macrophages were calculated from more than 20 fields per animal. Differences in the numbers of positively stained cells between two groups or among multiple groups were compared using the independent-samples *t*-test or one-way ANOVA, respectively. Experiments were conducted with six mice per group, and data are presented as mean ± SD. *P < 0.05 and **P < 0.01 vs. the *P. berghei* ANKA-infected control mice at 8 dpi; ^#^*P* < 0.05 and ^##^*P* < 0.01 vs. the *P. berghei* ANKA-infected control mice at 15 dpi. NS = non-significant, *P* > 0.05, relative to naïve mice

As shown in Fig. 6, immunohistochemical staining revealed that only a few or rare pulmonary CD68^+^ (a total macrophage marker), CD86^+^ (a M1-subtype macrophage marker), and CD206^+^ (a M2-subtype macrophage marker) macrophages were observed in the uninfected mice in the naïve, DSF, and TTM groups. However, the numbers of pulmonary CD68^+^, CD86^+^, and CD206^+^ macrophages were significantly elevated in the experimental MA-ALI/ARDS mice at 8 and 15 dpi relative to the naïve mice (*P* < 0.01). Notably, treatment of infected mice with DSF led to a significant increase in the numbers of pulmonary CD68^+^ macrophages (*P* < 0.01) and CD86^+^ macrophages (*P* < 0.01) relative to the *Pb* group at 8 and 15 dpi, but resulted in a dramatic decrease in the number of pulmonary CD206^+^ macrophages (*P* < 0.01). Conversely, the administration of TTM significantly reduced the numbers of pulmonary CD68^+^ (*P* < 0.05) and CD86^+^ macrophages (*P* < 0.05) in the experimental MA-ALI/ARDS mice relative to those in the *Pb* group, while dramatically elevating the number of pulmonary CD206^+^ macrophages (*P* < 0.01). Thus, our data indicate that treatment with DSF or TTM promoted or inhibited pulmonary macrophage M1 polarization in the process of MA-ALI/ARDS, respectively.

### Changes in the numbers of SLC31A1^+^-CD68^+^ and FDX1^+^-CD68^+^ pulmonary macrophages in experimental MA-ALI/ARDS mice after DSF or TTM treatment

**Fig. 7:**
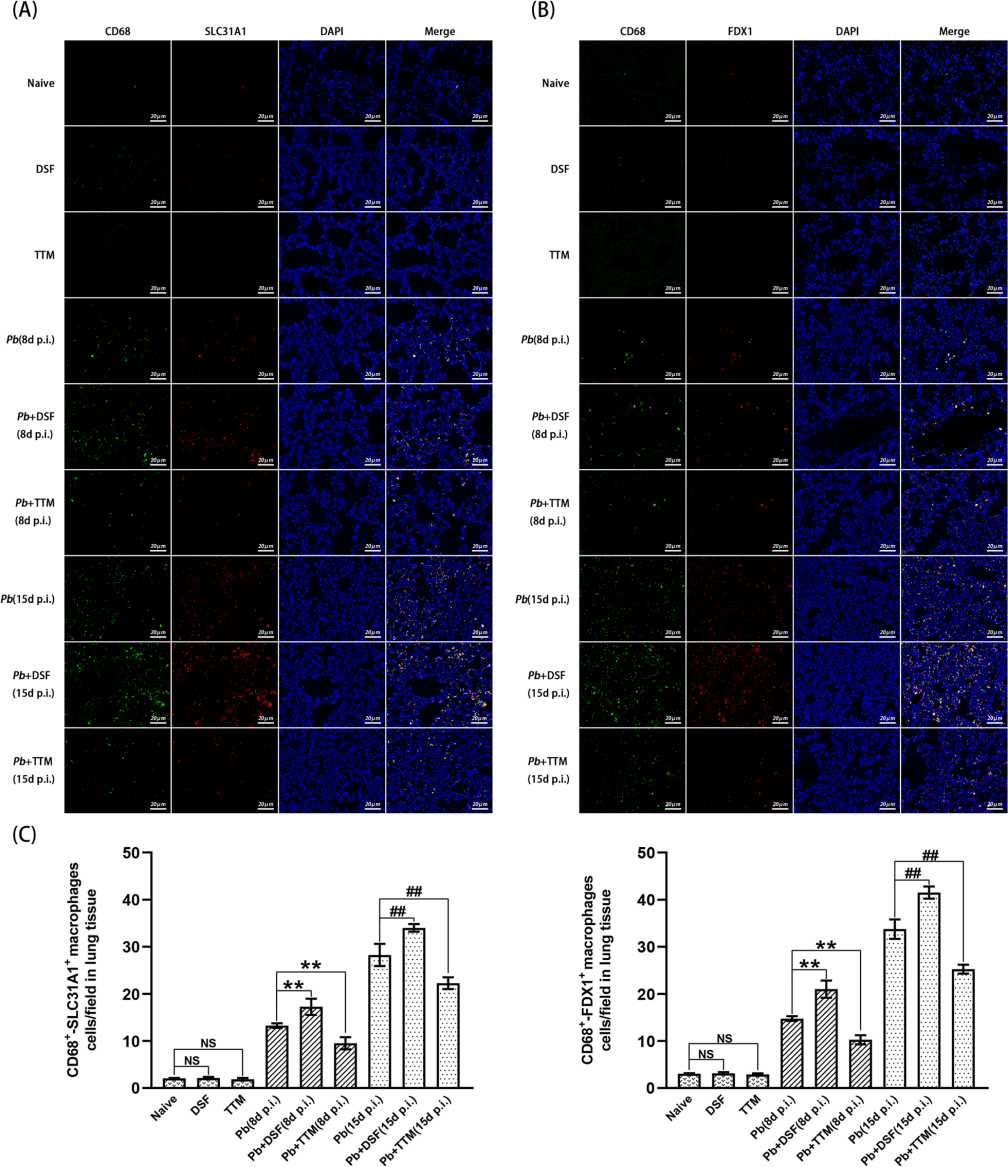
The effect of DSF or TTM on changes in the numbers of SLC31A1^+^-CD68^+^ and FDX1^+^-CD68^+^ pulmonary macrophages in experimental MA-ALI/ARDS mice. **A**, **B** Representative images of pulmonary SLC31A1^+^-CD68^+^ and FDX1^+^-CD68^+^ macrophages in different groups using double immunofluorescence staining under a fluorescence microscope at a magnification of ×200. The positively stained CD68^+^ macrophages appear green, while the positively SLC31A1^+^ or FDX1^+^ cells appear red. Co-expression of SLC31A1^+^-CD68^+^ or FDX1^+^-CD68^+^ cells exhibits a yellow color. **C** The numbers of positively SLC31A1^+^-CD68^+^ or FDX1^+^-CD68^+^ pulmonary macrophages were calculated from more than 20 lung fields per animal. Differences in the numbers of SLC31A1^+^-CD68^+^ and FDX1^+^-CD68^+^ macrophages between two groups or among multiple groups were compared using the independent-samples *t*-test or one-way ANOVA, respectively. Experiments were conducted with six mice per group, and data are presented as mean ± SD. *P < 0.05 and **P < 0.01 vs. the *P. berghei* ANKA-infected control mice at 8 dpi; ^#^*P* < 0.05 and ^##^*P* < 0.01 vs. the *P. berghei* ANKA-infected control mice at 15 dpi. NS = non-significant, *P* > 0.05, relative to naïve mice

Double immunofluorescence staining showed that rare or a few SLC31A1^+^-CD68^+^ and FDX1^+^-CD68^+^ pulmonary macrophages were observed in the uninfected mice in the naïve, DSF, and TTM groups (Fig. 7). However, *P. berghei* ANKA infection dramatically increased the numbers of SLC31A1^+^-CD68^+^ and FDX1^+^-CD68^+^ pulmonary macrophages in the *Pb* group at 8 and 15 dpi relative to those in the naïve group (*P* < 0.01). When compared with the *Pb* group, the numbers of SLC31A1^+^-CD68^+^ and FDX1^+^-CD68^+^ pulmonary macrophages were dramatically upregulated in the *Pb* + DSF group (*P* < 0.01), but were notably downregulated in the *Pb* + TTM group (*P* < 0.01), respectively. Collectively, our data indicate that the administration of DSF or TTM increased or decreased the numbers of SLC31A1^+^-CD68^+^ and FDX1^+^-CD68^+^ pulmonary macrophages in the process of MA-ALI/ARDS, respectively.

### Changes in the pulmonary inflammatory responses in experimental MA-ALI/ARDS mice after DSF or TTM treatment

**Fig. 8:**
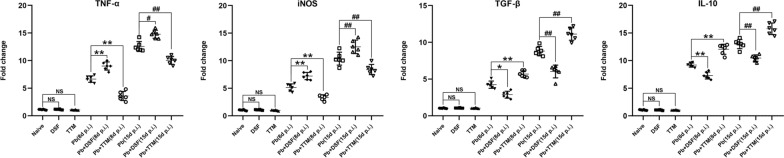
The effect of DSF or TTM on changes in pulmonary inflammatory response in experimental MA-ALI/ARDS mice. Total RNA was extracted from the lung tissue of the different groups, and the mRNA levels of TNF-α, iNOS, TGF-β, and IL-10 were determined using qPCR and 2^−ΔΔCT^ methods. Independent-samples *t*-tests or one-way ANOVA were conducted to assess differences between two groups or among multiple groups, respectively. Experiments were conducted with six mice per group, and data are presented as mean ± SD. *P < 0.05 and **P < 0.01 vs. the *P. berghei* ANKA-infected control mice at 8 dpi; ^#^*P* < 0.05 and ^##^*P* < 0.01 vs. the *P. berghei* ANKA-infected control mice at 15 dpi. NS = non-significant, *P* > 0.05, relative to naïve mice

As shown in Fig. 8, treatment with DSF or TTM had no remarkable effect on mRNA levels of tumor necrosis factor (TNF-α), inducible nitric oxide synthase (iNOS), transforming growth factor beta (TGF-β), or interleukin 10 (IL-10) in lung tissue from naïve mice (*P* > 0.05), while *P. berghei* ANKA infection led to dramatically elevated mRNA levels of all examined cytokines in lung tissue in the *Pb* group relative to levels in the naïve group (*P* < 0.01). Notably, the administration of DSF in the infected mice triggered higher mRNA levels of pro-inflammatory cytokines [TNF-α (*P* < 0.01 and *P* < 0.05) and iNOS (*P* < 0.01)] but lower mRNA levels of anti-inflammatory cytokines [TGF-β (*P* < 0.05 and *P* < 0.01) and IL-10 (*P* < 0.01)] in the lung tissues of the *Pb* + DSF group relative to the *Pb* group at 8 and 15 dpi, respectively. Conversely, mRNA levels of TNF-α (*P* < 0.01) and iNOS (*P* < 0.01) were downregulated but TGF-β (*P* < 0.01) and IL-10 (*P* < 0.01) levels were upregulated in the lung tissue of the *Pb* + TTM group relative to the *Pb* group at 8 and 15 dpi, respectively. Thus, our findings suggest that treatment with DSF can promote an excessive pro-inflammatory response in MA-ALI/ARDS, while TTM treatment can trigger an anti-inflammatory response.

### Changes in the mRNA levels of cuproptosis- and M1/M2 polarization-related markers in iRBC-stimulated RAW 264.7 cells after DSF-CuCl_2_ or TTM-CuCl_2_ treatment

**Fig. 9:**
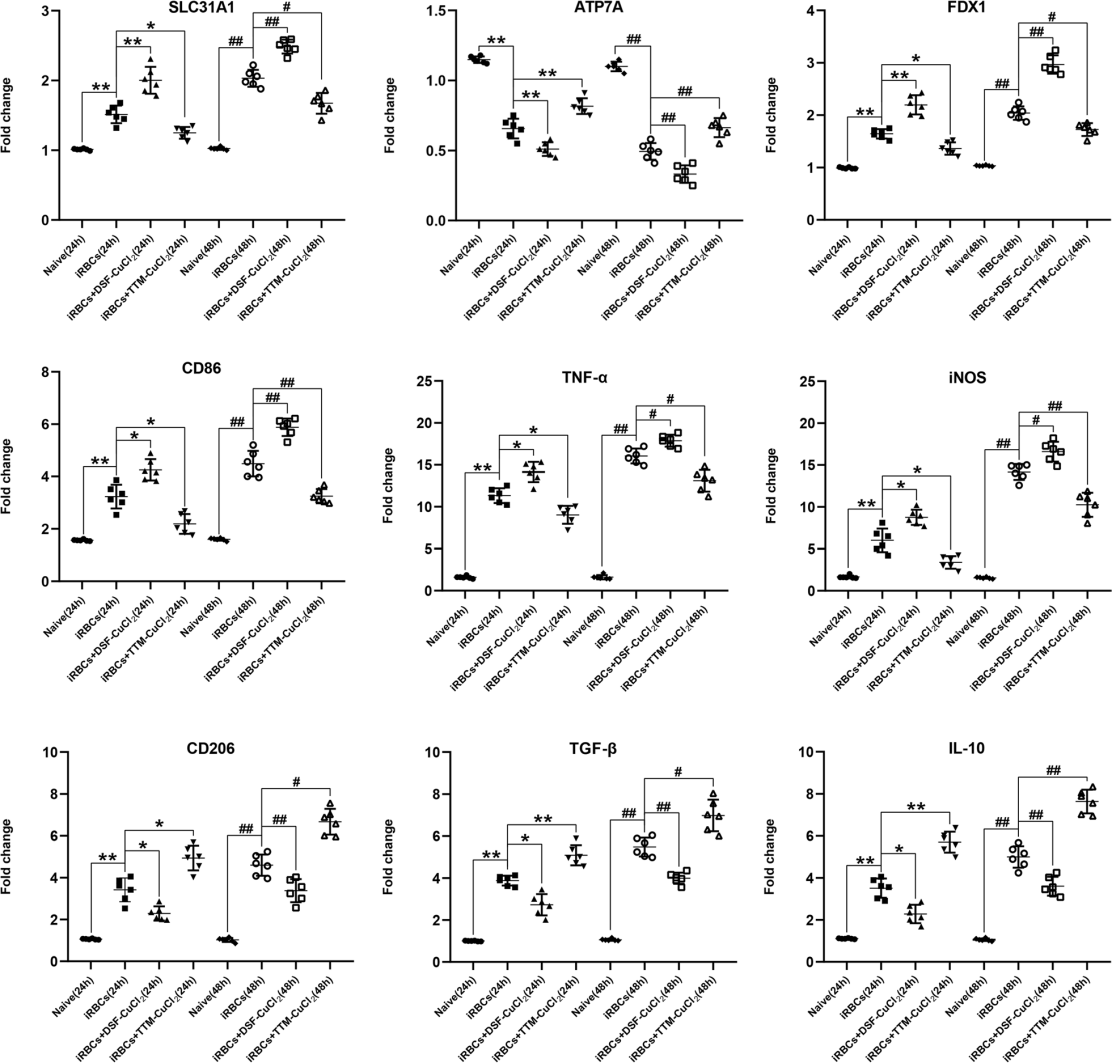
The effect of co-culture of DSF-CuCl_2_ or TTM-CuCl_2_ on the mRNA levels of SLC31A1, ATP7A, FDX1, CD86, CD206, TNF-α, iNOS, TGF-β, and IL-10 in iRBC-stimulated RAW 264.7 cells in vitro. The RAW 264.7 cells were pretreated with 5.0 × 10^6^ iRBCs for 6 h, then treated with PBS, DSF-CuCl_2_, or TTM-CuCl_2_ for 24 or 48 h. The qPCR method was used to quantify the mRNA levels of target genes. ^&^*P* < 0.05 and ^&&^*P* < 0.01 vs. RAW 264.7 cells treated with only PBS for 24 h; *P < 0.05 and **P < 0.01 vs. iRBC-stimulated RAW 264.7 cells for 24 h; ^$^ *P* < 0.05 and ^$$^ *P* < 0.01 vs. RAW 264.7 cells treated with only PBS for 48 h; ^#^ *P* < 0.05 and ^##^ *P* < 0.01 vs. iRBC-stimulated RAW 264.7 cells for 48 h. Data are taken as mean ± SD. The experiment was conducted 4–5 times with similar results

As shown in Fig. 9, higher mRNA levels of SLC31A1, FDX1, CD86, CD206, TNF-α, iNOS, TGF-β, and IL-10, but lower mRNA levels of ATP7A were observed in the iRBC-stimulated RAW 264.7 cells relative to the RAW 264.7 cells in the blank group at 24 h and 48 h of co-culture (*P* < 0.01), respectively. Notably, DSF-CuCl_2_ treatment significantly increased the mRNA levels of SLC31A1 (*P* < 0.01), FDX1 (*P* < 0.01), CD86 (*P* < 0.05 and *P* < 0.01), TNF-α (*P* < 0.05), and iNOS (*P* < 0.05), while dramatically decreasing the mRNA levels of ATP7A (*P* < 0.01), CD206 (*P* < 0.05 and *P* < 0.01), TGF-β (*P* < 0.05 and *P* < 0.01), and IL-10 (*P* < 0.05 and *P* < 0.01) in the iRBC-stimulated RAW 264.7 cells. Conversely, the iRBC-stimulated RAW 264.7 cells treated with TTM-CuCl_2_ showed downregulated mRNA levels of SLC31A1 (*P* < 0.05), FDX1 (*P* < 0.05), CD86 (*P* < 0.05 and *P* < 0.01), TNF-α (*P* < 0.05), and iNOS (*P* < 0.05 and *P* < 0.01), but a significant increase in the mRNA levels of ATP7A (*P* < 0.01), CD206 (*P* < 0.05), TGF-β (*P* < 0.05 and *P* < 0.01), and IL-10 (*P* < 0.01). Thus, these results indicate that co-culture of DSF-CuCl_2_ can promote cuproptosis and M1 polarization of RAW 264.7 macrophages in vitro, while the administration of TTM-CuCl_2_ can reverse these effects.

## Discussion

MA-ALI/ARDS is a severe complication of *P. falciparum* malaria that is characterized by multiple pathophysiological features, including alveolar–capillary barrier disruption, endothelial injury, and vascular fluid leakage. Increasing evidence has demonstrated that pulmonary macrophage accumulation/infiltration and its imbalanced M1/M2 polarization are closely associated with the onset and progression of MA-ALI/ARDS [14, 32]. Given that copper exposure has been shown to increase the risk of malaria infection [27, 28], and copper accumulation-induced cuproptosis triggers M1 macrophage polarization [29, 30], it was reasonably hypothesized that cuproptosis could modulate the pathogenesis of MA-ALI/ARDS by targeting pulmonary macrophage M1/M2 polarization. Our data showed that copper accumulation and cuproptosis were observed in the lung tissue of the experimental MA-ALI/ARDS mice, and further demonstrated that the administration of DSF exacerbated cuproptosis, promoted pulmonary macrophage M1 polarization, and ultimately worsened the severity of MA-ALI/ARDS. Conversely, treatment with TTM alleviated the severity of MA-ALI/ARDS by partially inhibiting pulmonary cuproptosis and macrophage M1 polarization. Thus, our findings suggest that blockage of cuproptosis represents a potential therapeutic target for treating MA-ALI/ARDS.

A growing body of studies have focused on the role of copper or copper overload-induced cuproptosis in the pathogenesis of lung diseases, including chronic obstructive pulmonary disease and lung adenocarcinoma [19, 29, 33]. A few studies showed that malaria patients had a lower level of serum copper than those of healthy controls [34–36], and further indicated a negative correlation between the level of serum copper and the proliferation of the malaria parasite [37]. It was also reported that a higher level of serum copper was observed in Asian malaria patients relative to healthy controls [26]. Furthermore, it was reported that female BALB/c mice with *P. berghei* ANKA displayed an increase in serum copper similar to that seen with *C. albicans* [24]. Notably, the selective removal of copper by copper chelators (2,9-dimethyl-1,10-phenanthroline, hydrochloride) completely eliminated the ring forms of *P. falciparum* in RBCs [38]. Currently, the role of copper overload-induced cuproptosis in the pathogenesis of MA-ALI/ARDS is unknown. In the present study, we observed a higher copper overload in the lung tissue of experimental MA-ALI/ARDS mice by both RAC staining and ICP-MS, followed by a notable increase in the expression of SLC31A1 (a copper import transporter) and FDX1 (a key positive cuproptosis regulatory), but a significant decline in the expression of ATP7A (a copper export transporter), thereby indicating that cuproptosis could occur in the process of MA-ALI/ARDS. Studies have demonstrated that DSF, a potent copper carrier, induced cuproptosis in a variety of cells or tissues [19, 39], whereas TTM, a copper chelator, conversely mitigated cuproptosis [19]. Given the fact that a dose of DSF (50 mg/kg) or TTM (30 mg/kg) had been adopted to explore the potential effects of copper or cuproptosis on disease pathogenesis [39–41], we thus treated experimental MA-ALI/ARDS mice with DSF (50 mg/kg) or TTM (30 mg/kg) to verify the role of cuproptosis in the severity of MA-ALI/ARDS. Our data showed that MA-ALI/ARDS mice with DSF treatment displayed an elevation in lung copper overload and expression of SLC31A1 and FDX1, but a decline in the expression of ATP7A, followed by a higher parasite burden and lung histopathological injury. Strangely, these trends were rescued by the administration of TTM in the process of MA-ALI/ARDS. Meanwhile, an in vitro experiment showed that the mRNA levels of SLC31A1 and FDX1 were significantly upregulated in iRBC-stimulated RAW 264.7 cells targeted with DSF, while the mRNA level of ATP7A was significantly downregulated in RAW 264.7 cells after DSF treatment. Likewise, treatment with TTM conversely rescued these conditions in iRBC-stimulated RAW 264.7 cells. Additionally, upregulated cuproptosis-related genes (*LIAS* and *PDHB*) were found to be positively associated with the occurrence of pulmonary inflammation in a septic mouse model [42]. It was also reported that cuproptosis-related genes (*POR*, *SLC7A5*, and *STAT3*) or *N*^6^-methyladenosine (m^6^A) methylation were strongly involved in the pathological process of sepsis-induced cardiomyopathy [43, 44]. Notably, bioinformatics analysis further validated the relationship between cuproptosis and sepsis-associated ALI [45]. Thus, our data indicate that cuproptosis could be strongly associated with the severity of MA-ALI/ARDS.

Macrophage activation, which is partially triggered by an increase in mitochondrial copper(II), has emerged as a multifaceted mediator in the pathogenesis of ALI/ARDS [32, 46]. It was further reported that accumulation of intracellular copper could promote macrophage M1 polarization and induce an excessive pro-inflammatory response to inhibit bacterial infection [47]. Alginate cross-linked with copper effectively drove macrophage M1 polarization to release the massive pro-inflammatory cytokines [48]. Moreover, cuproptosis was positively correlated with M1-subtype macrophages in the pathological process of alcoholic hepatitis by bioinformatics and experimental verification [49]. Similarly, TFP-Cu (Cu ion-coordinated *Tremella fuciformis* polysaccharide) selectively inhibited the proliferation of K7M2 tumor cells by promoting cell cuproptosis and M1 polarization of RAW 264.7 cells [50]. In the present study, our data also showed that administration of DSF led to a substantial increase in the number of pulmonary CD68^+^ and CD86^+^ macrophages (M1 subtype) in experimental MA-ALI/ARDS mice. Similarly, the in vitro experiment indicated that iRBC-stimulated RAW 264.7 cells with DSF-CuCl_2_ treatment displayed higher mRNA levels of CD86 and pro-inflammatory cytokines (TNF-α, iNOS), but showed lower mRNA levels of CD206 and anti-inflammatory cytokines (TGF-β, IL-10). In contrast, treatment with TTM strangely reversed these changes in both MA-ALI/ARDS mice and iRBC-stimulated RAW 264.7 cells. A previous study demonstrated that the number of CD68^+^ macrophages was significantly elevated in response to high levels of cuproptosis states [51]. Remarkably, our data also demonstrated that the numbers of SLC31A1^+^-CD68^+^ and FDX1^+^-CD68^+^ pulmonary macrophages were significantly increased in infected mice with DSF treatment, but dramatically decreased with TTM treatment. On the contrary, another study indicated that *N*,*N*-dimethyldithiocarbamate as a bactericidal antibiotic against *Streptococcus pneumoniae* plus copper dramatically enhanced macrophage bactericidal efficiency in vitro [52]. Thus, our data suggest that treatment with DSF or TTM aggravates or alleviates the severity of MA-ALI/ARDS by partially promoting or inhibiting cuproptosis-induced macrophage M1 polarization, respectively. A previous study indicated that SLC31A1 (a copper influx transporter) participated in the regulation of M1/M2 macrophage polarization [53]. Unfortunately, the underlying mechanism of cuproptosis influencing macrophage M1/M2 polarization currently remains largely unknown.

Although the role of cuproptosis in the severity of MA-ALI/ARDS with respect to DSF or TTM treatment was confirmed in the present study, there are still some unanswered questions: (1) it was not fully elucidated why *P. berghei* ANKA infection triggered the elevation in pulmonary copper overload; (2) our data did not validate whether the onset and progression of MA-ALI/ARDS were selectively targeted by cuproptosis-induced macrophage M1 polarization; and (3) our raw data also did not accurately elucidate the underlying mechanism of cuproptosis-induced macrophage M1 polarization. Therefore, further work to resolve these questions could help to better understand the role of cuproptosis in mediating the pathogenesis of MA-ALI/ARDS.

## Conclusions

In summary, this study confirmed the involvement of cuproptosis in the severity of MA-ALI/ARDS. Our data showed an increase in copper overload and cuproptosis as well as the numbers of SLC31A1^+^-CD68^+^ and FDX1^+^-CD68^+^ pulmonary macrophages in the experimental MA-ALI/ARDS mice. Additionally, our findings demonstrate that treatment with DSF can worsen lung injury in the process of MA-ALI/ARDS by partially exacerbating pulmonary macrophage M1 polarization and excessive pro-inflammatory responses. Conversely, the administration of TTM can reverse these changes by inhibiting pulmonary macrophage M1 polarization and triggering anti-inflammatory responses. However, further in-depth work is needed to elucidate the role of cuproptosis in mediating pulmonary macrophage M1/M2 polarization and its impact on the severity of MA-ALI/ARDS. Our data suggest that blockage of cuproptosis could serve as a potential therapeutic target for treating MA-ALI/ARDS.

## Supplementary Information

Additional file 1: Supplementary Table 1. The primers of target genes for the qPCR method. F: Forward primer; R: Reverse primer

## Data Availability

No datasets were generated or analyzed during the current study.
