# Supplementary material for: Role of cuproptosis in mediating the severity of experimental malaria-associated acute lung injury/acute respiratory distress syndrome
Source: Parasit Vectors. 2024 Oct 19;17:433. doi: 10.1186/s13071-024-06520-1 (PMC11489997; doi:10.1186/s13071-024-06520-1)
Supplement: Supplementary file 1 — Additional file 1: Supplementary Table 1. The primers of target genes for the qPCR method. F: Forward primer; R: Reverse primer [file 13071_2024_6520_MOESM1_ESM.docx]

| Genes | Primers ^a^ |
| --- | --- |
| SCL31A1 | F: 5’-AACCACACGGACGACAACATTAC-3’  R: 5’-AAGTAGAAGGTCATAGGCATCATCATC-3’ |
| ATP7A | F: 5’-TGGCAAGGCAGAAGTAAGATATAACC-3’  R: 5’-TCATTCCTCTCACAACAAGTTCCAAG-3’ |
| FDX1 | F: 5’-CGGAGCGGGAACTGCCATC-3’  R: 5’-TCTCGCCATCTCGGTTCTTGAAG-3’ |
| CD86 | F: 5’-AGCACTATTTGGGCACAGAGAAAC-3’  R: 5’-GTGAAGTCGTAGAGTCCAGTTGTTC-3’ |
| CD206 | F: 5’-CCTGAACAGCAACTTGACCAATAATG-3’  R: 5’-GTTCTCCAGTAGCCATCAACATCC-3’ |
| TNF-α | F: 5’-GCCTCTTCTCATTCCTGCTTGTGG-3’  R: 5’-GTGGTTTGTGAGTGTGAGGGTCTG-3’ |
| iNOS | F: 5’-GACGAGACGGATAGGCAGAGATTG-3’  R: 5’-AACTCTTCAAGCACCTCCAGGAAC-3’ |
| TGF-β | F: 5’-GGAAGAATACACCACCAGCAGTC-3’  R: 5’-CACGGTAGCAGTAGAAGATGATGATG-3’ |
| IL-10 | F: 5’-GGACAACATACTGCTAACCGACTC-3’  R: 5’-TGGATCATTTCCGATAAGGCTTGG-3’ |
| P*b*A 18S rRNA | F: 5’-ACGGGGAGCAAGAGCAGTATTTC-3’  R: 5’-CCCCACCAAAACCTGCCTTTATTG-3’ |
| GADPH | F: 5’-ACGGCAAATTCAACGGCACAG-3’  R: 5’-ACACCAGTAGACTCCACGACATAC-3’ |

**Additional file 1: Table 1. Sequences of genes primers used for qPCR assay.**

Notes: ^a^ F, Forward primer; R, Reverse primer.
